# Postmortem Biopsies of the Lung, Heart, Liver, and Spleen of COVID-19 Patients

**DOI:** 10.7759/cureus.20734

**Published:** 2021-12-27

**Authors:** Isil Yurdaisik, Ahu S Demiroz, Aysim B Oz, Mustafa Akker, Aysegul Agirman, Suleyman Hilmi Aksoy, Fuad Nurili

**Affiliations:** 1 Radiology, Istinye University Medical Park Gaziosmanpasa Hospital, Istanbul, TUR; 2 Pathology, Cerrahpaşa Faculty of Medicine, Istanbul University, Istanbul, TUR; 3 Intensive Care Unit, Istinye University Medical Park Gaziosmanpasa Hospital, Istanbul, TUR; 4 Radiology, Dr. Siyami Ersek Thoracic and Cardiovascular Surgery Training and Research Hospital, Istanbul, TUR; 5 Radiology, Galata University, Istanbul, TUR; 6 Radiology, Memorial Sloan Kettering Cancer Center, New York, USA

**Keywords:** spleen, sars-cov-2, postmortem, lung, liver, histopathologic, heart, covid-19, biopsy

## Abstract

Objective

We aimed to evaluate histopathologic alterations in the lung, heart, liver, and spleen of coronavirus disease 2019 (COVID-19) decedents through postmortem core needle biopsies.

Materials and methods

Patients who died of reverse transcription-polymerase chain reaction-proven COVID-19 were included in this postmortem case series. Postmortem percutaneous ultrasound-guided biopsies of the lungs, heart, liver, and spleen were performed using 14- and 16-gauge needles. Biopsy samples were stained with hematoxylin-eosin and examined under a light microscope. Clinicodemographic characteristics, chest computed tomography (CT) images, and COVID-19-related treatments of the patients were also collected.

Results

Seven patients were included in this study. Liver and heart tissue samples were available from all patients, and lung and spleen tissue samples were available from five and three patients, respectively. Chest CT images predominantly revealed bibasilar ground-glass opacities. Lung biopsies showed diffuse alveolar damage in all biopsy specimens. Heart findings were nonspecific and largely compatible with the underlying disease. Patchy necrosis, steatosis, and mononuclear cell infiltration were the main findings in the liver biopsies. Splenic histopathological examination showed that splenic necrosis and neutrophil infiltration were common findings in all patients.

Conclusion

Tissue acquisition was complete for the heart and liver and acceptable for the lungs. The amount of tissue was sufficient for a proper histopathologic examination. Histopathological findings were generally in accordance with previous autopsy studies. Radiological findings of the lung were also correlated with the histopathologic findings. We consider that a postmortem biopsy is a feasible alternative for histopathological examinations in COVID-19 decedents.

## Introduction

A novel virus called severe acute respiratory syndrome coronavirus 2 (SARS-CoV-2) first appeared in China through rapidly progressing pneumonia cases and then gave rise to a pandemic. At the time of writing this manuscript, coronavirus disease 2019 (COVID-19) has claimed more than 720,000 lives around the world [1]. The pandemic is still ravaging many parts of the world, and fears of a more widespread second wave are growing.

The exact pathophysiology of COVID-19, the disease caused by SARS-CoV-2, has yet to be fully elucidated. According to preliminary studies, the virus enters the cells via angiotensin-converting enzyme 2 (ACE2) cell surface receptors [2,3]. As these receptors are abundant in lung tissue, the disease affects the lungs more severely than other parts of the body. As in the case of other newly described disease entities, histopathological examination of affected tissues is of paramount importance to better understand the pathophysiology of COVID-19. However, with the fear of spreading the disease, the autopsy of COVID-19 victims is prohibited or advised against, even in developed countries [4]. This reluctance to perform autopsies translates to the relative scarcity of autopsy studies, which are not commensurate with the commonality of COVID-19. This approach has been criticized by some authors [4,5]. As a result of the efforts of the scientific community, albeit still insufficient, the number of autopsy studies on COVID-19 patients has increased slightly in recent literature [6-11].

One way to overcome the regulatory barriers against performing an autopsy and the fear of contraction is postmortem biopsy. As the operator spends less time performing the procedure and with less exposure to the tissues of the decedent, unlike in autopsy, postmortem biopsies seem to be a reasonable and acceptable alternative to the autopsy of COVID-19 patients. However, as in the case of autopsies, postmortem biopsy studies are not sufficient during the COVID-19 pandemic [12-14].

So far, the number of postmortem biopsy studies is limited in the literature, especially on organs other than the lung. Thus, we aimed to perform a case series in which postmortem core needle biopsies of the lung, liver, heart, and spleen of COVID-19 decedents were conducted.

## Materials and methods

Seven patients who died of COVID-19 were included in this postmortem study. All patients had confirmed SARS-CoV-2 infection through the reverse transcription-polymerase chain reaction (RT-PCR) test applied to samples obtained with nasopharyngeal and oropharyngeal swabs. The RT-PCR tests (Bio-Rad, Life Science Research, Hercules, CA) were performed by trained technicians according to the manufacturer’s guidelines. All patients with suggestive symptoms of COVID-19 were admitted to the dedicated COVID-19 ward of our pandemic hospital. Before the beginning of the study, the necessary approval was obtained from the Istinye University Clinical Research Ethics Committee (2017-KAEK-120/2/2020.G-039). The written informed consent forms were signed by the legal guardians of all included patients before the postmortem biopsy.

One of the authors (IY) performed ultrasound-guided multiple percutaneous biopsies of the lungs, kidneys, heart, and spleen using 14- and 16-gauge biopsy needles.

The tissue samples were fixed in 10% buffered formalin for 48 hours and processed for routine histopathological examination. The samples were then embedded in paraffin for serial sectioning. Sections 4 μm in size were stained with hematoxylin and eosin (H&E) and examined under a light microscope by experienced pathologists.

The electronic hospital database system and patient charts were reviewed to obtain data regarding history, presenting symptoms and physical findings, length of hospital stay (in the ward and intensive care unit [ICU]), administered treatments, admission laboratory values, and imaging studies for each study patient.

Statistical analysis

Descriptive analyses were summarized as the median (range) and percentage as appropriate. All statistical analyses were performed using SPSS version 24 (IBM Corp, Armonk, NY) statistical software package.

## Results

Laboratory and clinical characteristics of the patients

Seven deceased COVID-19 patients (two females) were included in this postmortem biopsy study. The median age was 62 years (range: 39-93 years). All patients had comorbid chronic medical conditions. Hypertension was the most common comorbidity and was present in all patients, followed by diabetes mellitus in five of the seven patients. One patient died in the ward, and the remaining patients died in the ICU. As part of the national guideline issued by the Ministry of Health of Turkey, all patients were treated with the following drug protocol: azithromycin + hydroxychloroquine + favipiravir. Depending on the severity of the disease and at the caring physician’s discretion, some patients were treated with tocilizumab and/or convalescent plasma donated by COVID-19 survivors. Patients who were connected to a mechanical ventilator in the ICU were administered 1 mg/kg body weight of methylprednisolone. Patients’ demographic and clinical characteristics are shown in Table 1.

**Table 1:** Clinical and demographic characteristics and chest CT findings of the study patients. A: azithromycin; F: female; Fa: favipiravir; M: male; CABG: coronary artery bypass graft; CAD: coronary artery disease; COVID-19: coronavirus disease 2019; DM: diabetes mellitus; GGO: ground-glass opacities; H: hydroxychloroquine; HT: hypertension; LVH: left ventricular hypertrophy; MP: methylprednisolone; RLL: right lower lobe; RML: right middle lobe; T: tocilizumab.

Patient	Sex, age	Comorbid conditions	Length of hospital stay (days)	Length of ICU stay (days)	Length of mechanical ventilation (days)	Time between the last chest CT scan and death (days)	Chest CT findings	COVID-19 treatment
Patient 1	M, 62	CABG, CAD, LVH, HT	9	0	0	-	-	A + H
Patient 2	M, 39	DM, HT, LVH	22	4	0	3	GGO prominent in RLL, cardiomegaly	A + H + Fa + MP
Patient 3	F, 66	CAD, LVH, HT	19	7	7	5	Bilateral extensive GGO, consolidation in basal areas, cardiomegaly, pericardial effusion	A + H + Fa + T + MP + plasma
Patient 4	F, 59	CAD, LVH, HT, DM	23	14	19	3	GGO, consolidation, subsegmental atelectasis and 4 cm pleural effusion, cardiomegaly, pericardial effusion	A + H + Fa + T + MP + plasma
Patient 5	M, 93	CAD, LVH, HT, DM	1	21	17	5	GGO in RLL, consolidation in the left lung, left diaphragm elevation, cardiomegaly, pleural effusion	A + H + Fa + T + MP + plasma
Patient 6	M, 55	DM, LVH	20	5	3	4	Bibasilar and RML GGO, cardiomegaly, pericardial effusion	A + H + Fa + MP
Patient 7	M, 75	CAD, DM	19	6	5	3	Bibasilar GGO more prominent on right side, pleural and pericardial effusion, cardiomegaly	A + H + Fa + MP

Chest CT findings

**Figure 1:**
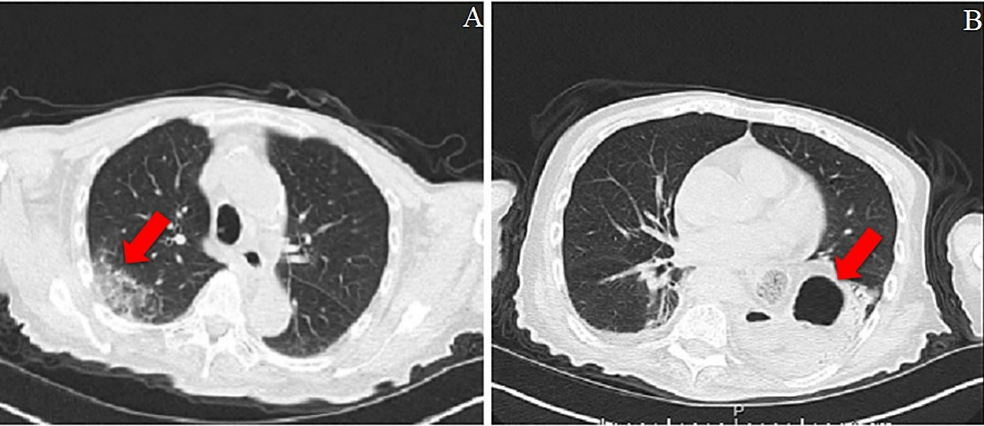
Chest CT scan of a 93-year-old male patient who died five days after his CT scan. A. COVID-19 pneumonia. B. Left-sided hiatal herniation.

**Figure 2:**
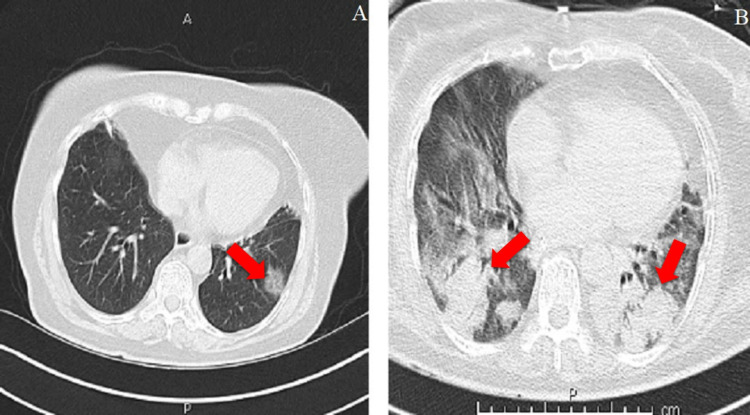
Chest CT scan of a 66-year-old female patient. A. Third chest CT image taken 14 days after the first and seven days after the second CT showing increased GGO in the upper lobes. B. Air bronchograms and GGO accompanying consolidations in the lower lobes. GGO: ground-glass opacities.

Six of the seven patients underwent chest CT scanning. The universal finding in all patients was the presence of ground-glass opacities (GGO), which were usually more prominent in the basilar areas of the lungs. Five patients (55.5%) had pleural effusion, and three patients (33.3%) had pericardial effusion (one patient had simultaneous pleural and pericardial effusion, and one had neither of them). One notable feature was that all patients had cardiomegaly on the CT images. Given the prevalent history of coronary artery disease and left ventricular hypertrophy among the patients, this latter finding was not surprising. Table 1 presents a summary of the chest CT imaging findings. Figures 1, 2 illustrate the lung radiological findings in two patients.

Histopathologic findings

Lungs

Lung tissue was available in five of the seven patients. One patient (Patient 4) showed findings of early acute respiratory distress syndrome (ARDS). This patient had hyaline membranes and edema in the alveoli. In all other patients (Patients 2, 3, 5, and 7), examination of the lung biopsy specimens revealed the organizing phase of ARDS. These patients had remarkable septal thickening and collagen increase. Table 2 presents the details of the histopathologic findings of the lung biopsies.

**Table 2:** Pulmonary pathologic findings of patients who died from COVID-19. ARDS: acute respiratory distress syndrome; COVID-19: coronavirus disease 2019; DAD: diffuse alveolar damage; NI: not identified.

Lung pathologic findings	Patient 1	Patient 2	Patient 3	Patient 4	Patient 5	Patient 6	Patient 7
Interstitial/alveolar edema	No lung tissue	NI	Present	NI	NI	No lung tissue	NI
Capillary microthrombi	-	Present	NI	Present	NI	-	NI
Desquamation	-	Present	Present	Present	Present	-	Present
Hyaline membrane formation	-	Present	Present	Present	Present	-	NI
Hyaline membrane organization	-	NI	NI	Present	NI	-	NI
Type II pneumocyte proliferation	-	Present	Present	NI	Present	-	Present
Cytopathic effect	-	Present	Present	NI	NI	-	NI
Interstitial cell increase	-	Present	Present	Present	Present	-	Minimal
Septal collagen increase	-	NI	NI	NI	NI	-	Present
Septal thickening	-	Present	Present	Present	Present	-	Present
Mononuclear cell increase	-	Minimal	Present	NI	Minimal	-	NI
Pathologic diagnosis	-	DAD (epithelial and vascular)	DAD (epithelial)	DAD (epithelial and vascular)	DAD (epithelial)	-	DAD (vascular and fibrotic)

**Figure 3:**
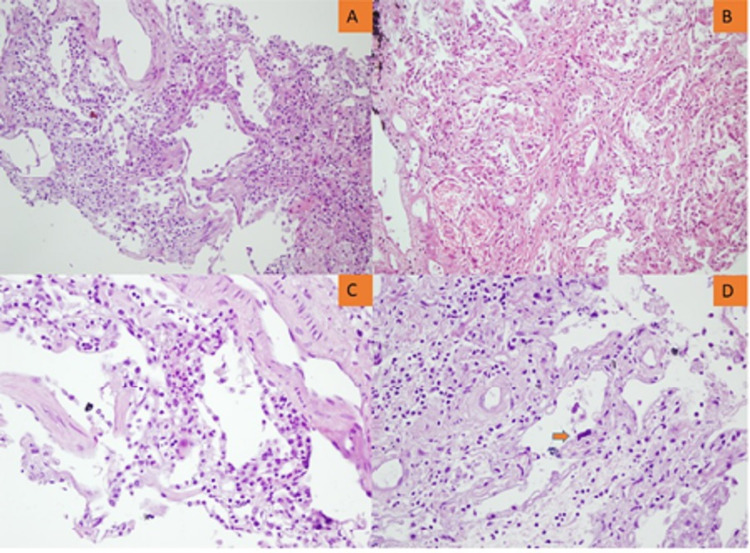
Lung microscopy showing the late organizing phase of ARDS. A. Septal thickening with interstitial cellular increase (x100). B. Septal thickening with interstitial cellular and collagenous increase (x200). C. Interstitial inflammatory cellular infiltration (x400). D. Interstitial thickening with cellular and collagenous increase, atypical type II pneumocyte (orange arrow). ARDS: acute respiratory distress syndrome.

**Figure 4:**
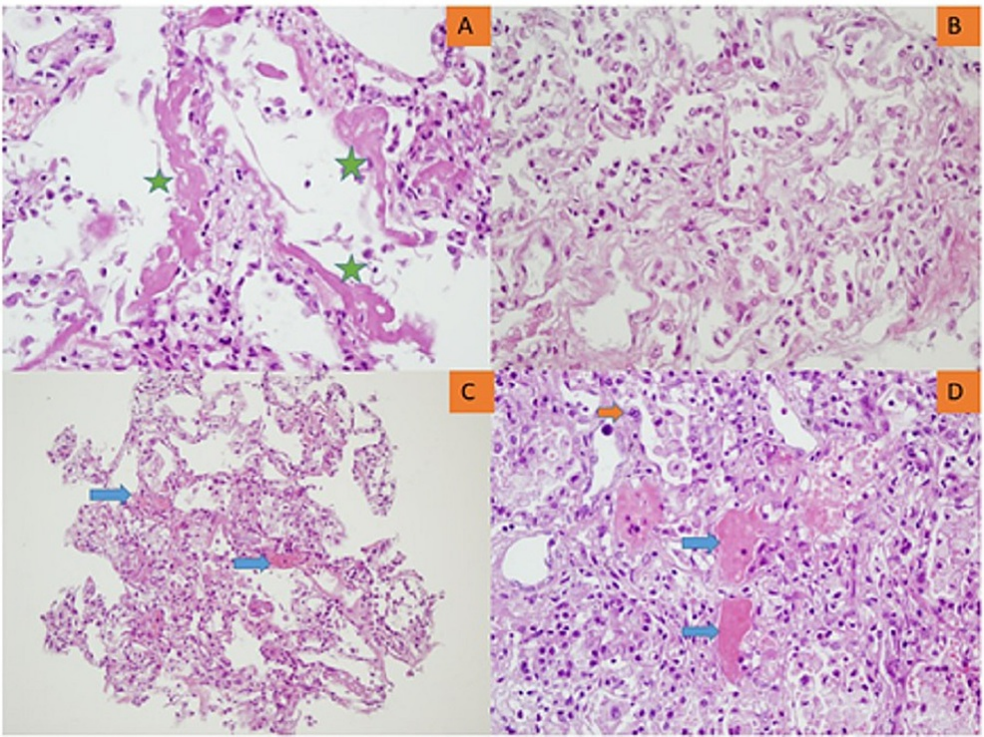
Lung microscopy showing the early phase of ARDS. A. Hyaline membrane (green asterisk, x400). B. Type II pneumocyte proliferation and alveolar desquamation (x400). C. Microthrombi (blue arrows, x200). D. Microthrombi (blue arrows), type II pneumocyte proliferation, atypical type II pneumocyte (orange arrow), and alveolar desquamation (x400). ARDS: acute respiratory distress syndrome.

Figures 3, 4 illustrate the epithelial and/or vascular diffuse alveolar damage, respectively.

Heart

Heart tissue was present in the biopsy samples of all patients. Only one patient had completely normal cardiac histopathological findings (Patient 3). Cardiomyocyte hypertrophy was the most common finding among the other patients. Glycogen increase (Patients 1, 4, and 5) and fibrosis (Patients 2 and 6) were also observed (Table 3).

**Table 3:** Pathologic findings in the heart, liver, and spleen of deceased COVID-19 patients.

Patient	Heart	Liver	Spleen
1	Hypertrophy, glycogen increase	Patchy large necrosis areas, parenchymal neutrophil infiltration, bile in hepatocytes (stasis findings)	No splenic tissue
2	Mild fibrosis, hypertrophy	Microthrombi, extensive hepatocyte necrosis, portal and lobular mononuclear cell increase, macrovesicular steatosis	No splenic tissue
3	Normal histologic findings	Extensive macrovesicular steatosis, mild sinusoidal dilation, bile pigment in hepatocytes, patchy necrosis, focal neutrophil infiltration	No splenic tissue
4	Lipomatosis, glycogen increase, hypertrophy	Patchy necrosis, macrovesicular steatosis	Neutrophils in the red pulp, focal necrosis
5	Hypertrophy, glycogen increase	Extensive hepatocyte necrosis, mononuclear, and a few neutrophil infiltrations	No splenic tissue
6	Fibrosis	Patchy necrosis	Neutrophils in the red pulp, focal necrosis
7	Hypertrophy	Extensive macrovesicular steatosis, mild mononuclear cell increase in the portal areas	Neutrophils in the red pulp, focal necrosis

Liver

Liver tissue was present in all biopsy samples. Hepatocyte necrosis was present in all but one patient. Patients 2 and 5 had extensive hepatocyte necrosis, and the other patients (Patients 1, 3, 4, and 6) had patchy necrosis. Patients 2, 3, 4, and 7 had macrovesicular steatosis. Two patients (Patients 1 and 3) had neutrophil infiltration in their liver parenchyma, and the other three patients had mononuclear cell infiltration (Patients 2, 5, and 7). The liver histopathologic findings of the patients are presented in Table 3.

Spleen

Splenic tissue was available in only three patients. Examination of all biopsy samples revealed neutrophils in the red pulp and necrosis (Table 3).

## Discussion

The notable findings of this case series are as follows: (i) tissue acquisition through ultrasound-guided postmortem biopsies was acceptable, particularly for the liver and heart. A sufficient amount of lung tissue was obtained in five of the seven patients. (ii) The universal radiological finding was the presence of GGO, particularly in the lower lung lobes. The pathologic counterpart of these images was the organizing phase of ARDS. All but one patient had hyaline membranes in their alveoli. (iii) This is the first study to report splenic postmortem biopsy results in COVID-19 patients. The common finding was necrosis and neutrophil infiltration in the red pulp.

Several studies and case reports have been published to understand histopathological changes and acquire possible ultrastructural clues underlying the widespread damage inflicted by SARS-CoV-2. As the lungs are the main target of the virus, pulmonary imaging and histopathology have attracted much attention. Three rough stages have been defined for COVID-19 [15]. The earliest phase is SARS-CoV-2 infection, characterized by fever, dyspnea, and other flu-like symptoms. The second stage involves viral pneumonia, which causes pulmonary inflammation and coagulopathy. Some cases progress to ARDS in this stage. The third and last stage is characterized by relieving symptoms and pulmonary fibrosis. These stages are not necessarily consecutive and can coexist in the same patient.

Pulmonary findings are histopathologically classified into three patterns [16-18]. The most common and earliest pattern is diffuse alveolar damage (DAD), which shows varying degrees of organization, desquamation, type 2 pneumocyte hyperplasia, and viral cytopathic changes. The second pattern (vascular) involves intra-alveolar fibrin deposition and/or microvascular thrombi. Fibrotic patterns include fibrotic DAD and/or interstitial fibrosis. A meta-analysis [19] evaluating autopsy studies revealed that pulmonary histopathologic examinations showed an epithelial pattern of injury in 85% of the included patients. When present, mononuclear cells were the predominant cell type infiltrating the lung interstitium. A vascular pattern was present in 59% of the patients, and a fibrotic DAD pattern was observed in 22% of the patients. Among the patients, 60% had more than one pattern concurrently. Our findings were generally in agreement with previous autopsy studies. The most common pulmonary injury pattern among our patients was epithelial DAD. It was present in four of the five patients (80%), isolated in two patients, and alongside a vascular DAD pattern in the other two patients. Interstitial inflammation was noted in three patients (50%) and was mononuclear in all cases. One patient had a concomitant fibrotic and vascular pattern.

Only a few studies to date have reported postmortem biopsy findings in COVID-19 decedents [12-14]. In these studies, 18 COVID-19 decedents were examined through postmortem biopsies. One study [13] evaluated only lung tissue, and two [12,14] studies also examined liver and heart tissue, in addition to lung tissue. Beigmohammadi et al. [12] performed postmortem biopsies with ultrasound guidance, whereas Tian et al. [14] performed blind biopsies based on reference points. Flikweert et al. [13] used ultrasound or CT guidance to perform postmortem biopsies. In these studies, the authors obtained sufficient lung tissue for histopathological examination. Conversely, sufficient lung tissue was not obtained from the two patients in the present study.

Similar to our study, Flikweert et al. and Tian et al. [13,14] also included chest radiological findings in their reports. Regarding pulmonary histopathologic findings, diffuse alveolar damage was the predominant finding in all three studies.

Myocardial injury is prevalent in COVID-19 patients hospitalized in ICU [20-22]. In autopsy studies, no specific pathological finding associated with COVID-19 was found in more than half of the patients. Most of the cardiac histopathological findings could be explained by the underlying cardiovascular disease of the patients, such as coronary artery disease and heart failure [23,24]. In the other half, mild interstitial mononuclear cell infiltration, lymphocytic myocarditis, and myocardial edema were reported [19,25]. One remarkable aspect was that pericardial effusion was rare. Postmortem biopsy studies have reported nonspecific histopathological findings that are mostly compatible with the underlying cardiovascular disease of the patients [12,14]. In contrast to autopsy and postmortem biopsy studies, pericardial effusion was frequent in our series. Four of the six patients (66.6%) had varying degrees of pericardial effusion. However, no specific COVID-19-associated cardiac histopathologic findings were observed in any of the examined specimens. The observed changes were in accordance with the underlying cardiovascular diseases of the decedents.

Autopsy studies performed on patients who died of COVID-19 showed mild steatosis, patchy hepatic necrosis, and mild sinusoidal dilation in the vast majority of patients [23,26]. In their postmortem series, Beigmohammadi et al. [12] reported sinusoidal dilation, microvesicular and macrovesicular steatosis, and mild portal inflammation. Tian et al. [14] reported sinusoidal dilation and patchy hepatocyte necrosis. Our results showed patchy hepatocyte necrosis, mononuclear and/or neutrophil infiltration, and macrovesicular steatosis in the liver biopsy samples.

Autopsy studies have shown lymphocyte reduction and focal necrosis, infarction, and hemorrhage in the spleen [19,27]. None of the available postmortem studies have obtained splenic biopsies [12,14]. In our series, splenic tissue was available in three patients. Splenic tissue was not present in the four biopsy samples. All patients had focal necrosis and neutrophil infiltration in the red pulp.

Histopathologic changes in the liver, lung, heart, and spleen in the postmortem biopsy samples were generally similar to those obtained through autopsy. The tissue acquisition rates were quite high: 100% for the liver, 88.8% for the lung, and 88.8% for the heart. Thus, we believe that postmortem biopsies can provide comparably good histopathological results compared with autopsy studies. Furthermore, in postmortem studies, pulmonary histopathologic findings were concordant with radiological images.

This study has several limitations. First, although our sample was not smaller than that of other postmortem biopsy studies, examining more cases can yield more generalizable results. Second, we did not use immunohistochemical staining. Nevertheless, H&E staining was largely sufficient for the purposes of the study. Third, we did not examine the presence of SARS-CoV-2 in the biopsied tissues. These data, along with the ACE2 expression level, can shed more light on the pathophysiological changes seen in COVID-19. Lastly, as organ involvement could be patchy and as there was no chance of seeing the organs macroscopically, as in autopsy, postmortem biopsy studies have inherently a more limited tissue availability than autopsy studies. This might have affected our results to some extent.

## Conclusions

In conclusion, this study performed a postmortem biopsy series on COVID-19 decedents by sampling lung, heart, liver, and spleen tissues. This work is the first postmortem biopsy study to examine splenic core needle biopsies. An adequate amount of tissue for histopathologic examination was obtained in most biopsies. When adequate tissue was sampled, the results were generally consistent with previous autopsy studies. The pulmonary radiological findings were correlated with lung histopathologic examination results. A study design comparing postmortem biopsies with autopsy findings of the same patients can certainly determine the role of postmortem biopsies in COVID-19 decedents.
